# Understanding overactive bladder and urgency incontinence: what does the brain have to do with it?

**DOI:** 10.12688/f1000research.16418.1

**Published:** 2018-11-29

**Authors:** Ariana L. Smith

**Affiliations:** 1 Division of Urology, University of Pennsylvania , Philadelphia, PA, USA

**Keywords:** Overactive bladder, urgency urinary incontinence, neuroimaging, neuromodulation

## Abstract

Understanding the pathophysiologic mechanisms responsible for overactive bladder (OAB) and urgency urinary incontinence (UUI) is critical to advancing the treatment options available to men and women with this prevalent and bothersome condition. Development of novel technologies and advanced functional neuroimaging modalities has provided us with new information to support and refine existing mechanistic theories. Emerging research on central pathophysiologic mechanisms of OAB from functional magnetic resonance imaging may provide new targets for therapeutic interventions and opens the door for novel treatment strategies. Several regions of interest—specifically the anterior cingulate gyrus, insula, and frontal cortices—have been implicated as areas of activation in women with OAB, suggesting a neural correlate of the experience of urgency. The cerebellum and parietal lobe have demonstrated increased activation during inhibition of voiding, and increased connectivity between the cerebellum and parietal lobe and the right insula and anterior cingulate gyrus has been demonstrated in women with UUI compared with controls. Evolving literature is beginning to shed light on the prerequisite effective connections between regions of interest in the healthy bladder and negative connectivity in OAB and UUI. Precision medicine with individualized care pathways may better select available treatment modalities for rightful recipients, thus improving efficacy with prescribed treatment approaches and adherence to therapy.

## Introduction

Overactive bladder (OAB) with or without urgency urinary incontinence (UUI) is a prevalent and costly condition that greatly impairs quality of life in men and women ^1, 2^. Treatment often involves multifaceted therapy without identification or correction of the underlying pathophysiologic mechanism for the condition. Pharmacologic therapy, the mainstay of treatment, in the form of antimuscarinic or beta agonist agents ostensibly works at the level of the end organ, the bladder smooth muscle. It can be costly and often results in bothersome side effects with only a marginal improvement in symptoms and poor persistence on therapy ^3, 4^. Neuromodulation is a treatment modality used in patients refractory to medication and, in the sacral application, is thought to modulate aberrant afferent signaling between the bladder and the brain ^5^. Botulinum toxin injection in the bladder modulates neurotransmission through complex inhibitory effects on neurotransmitters, neuropeptides, and receptors such as that of acetylcholine ^6^. Despite its many successes, not all patients respond or have sustained response to available forms of neuromodulation and these therapies have complications. Additional options are needed to treat the estimated 29.8 million cases of OAB among men and women in the United States ^7^. Several interconnected brain centers have been implicated in controlling bladder function and dysfunction. Changes such as neuronal loss, demyelination, age-related volume loss, abnormal afferent signaling, and altered neurotransmitter release have been related to OAB ^8^. Emerging research on central pathophysiologic mechanisms of OAB from functional magnetic resonance imaging (fMRI) may provide new targets for therapeutic interventions and opens the door for novel treatment strategies. This article will look at the current understanding of the brain–bladder connection and incorporate recent evidence from fMRI studies.

## Mechanisms of bladder control

OAB and UUI result from the inability of the bladder to comfortably and appropriately store urine. OAB may be the outcome of increased bladder sensation during bladder filling or may be caused by involuntary bladder contractions, termed detrusor overactivity. Although the cause is likely multifactorial, aberrant interactions between the central and peripheral nervous systems and the end organ (the bladder) have been implicated in the absence of overt neurologic disease ^9^. Therapy is aimed at decreasing sensory (afferent) input, decreasing involuntary (efferent) contractions, and increasing bladder capacity. Antimuscarinic medications have long been used to treat the symptoms of OAB and UUI and were historically presumed to work via blockade of muscarinic receptors in the bladder to inhibit smooth muscle contraction ^10^. More recent studies have identified muscarinic receptors in the bladder urothelium, the spinal ganglia, and the brain, thus expanding the potential site of activity beyond the bladder smooth muscle ^11– 13^.

## Functional neuroimaging

Blood oxygen level-dependent (BOLD) fMRI is a functional neuroimaging modality that has emerged as a useful tool in the study of the central pathways of micturition and bladder dysfunction ^14^. The BOLD signal detected by fMRI reflects cerebral blood flow discriminately to distinct regions of the brain, an indirect measure of neuronal activity in that region. Several regions of interest (ROIs) in the central nervous system have been identified as being involved in the healthy and diseased micturition processes through comparison of BOLD activity during a relevant specific task such as voiding or experiencing urgency. These areas are felt to be the centers that generate the sensation of urgency and the accompanying emotional response, motor output, and executive function to execute voiding ^15– 18^. Specifically, the anterior cingulate gyrus, insula, and frontal cortices have been implicated as areas of increased activation in women with OAB ^19, 20^. Furthermore, studies using bladder filling to provoke urgency produce the most striking brain responses, suggesting a neural correlate of the experience of urgency ^21^. The cerebellum and parietal lobe have demonstrated increased activation during inhibition of voiding and contraction of the pelvic floor muscles ^22^. Increased connectivity between the cerebellum and parietal lobe and the right insula and anterior cingulate gyrus has been demonstrated in women with UUI compared with controls ^23^. Connectivity represents the complex functional interconnection of spatially distinct neuronal networks as an integrated system. Evolving literature is beginning to shed light on the prerequisite effective functional connections between ROIs in the healthy bladder and negative or poor connectivity in OAB and UUI ^23, 24^. The primary limitation of fMRI in the assessment of bladder function is the fair to poor test-retest repeatability, which, though comparable to the repeatability of other fMRI studies with repeated tasks, yields too much variability to provide insight on an individual patient level ^25^.

## Abnormal brain region activity

Therapy aimed at decreasing abnormal activity in implicated areas of the brain has proof of principle ^26, 27^. Pontari *et al.* studied brain activity changes by using 1.5 Tesla fMRI in women with OAB who were randomly assigned to an antimuscarinic (tolterodine) or placebo ^27^. The authors used bladder filling-induced urgency as the study task. Mirroring the well-known large placebo effect seen in pharmacologic studies, this study demonstrated a significant improvement in voiding symptoms in both the treatment and the placebo arms with similar reductions in frequency, urgency, and incontinence episodes. Both groups demonstrated less brain activation globally on fMRI with a full bladder after treatment. However, activation and deactivation data indicated differences in brain activity in participants taking tolterodine compared with placebo. Specifically, greater activation was seen in the parietal cortex and greater deactivation in the cerebellum was seen in the tolterodine group compared with placebo.

Weissbart *et al.* (the author’s group) studied changes in brain activity by using 1.5 Tesla fMRI in women with refractory OAB before and after sacral nerve stimulation ^26^. In that study, the neurostimulator unit was turned off immediately prior to imaging and bladder filling-induced urgency was used as the study task. We found that in women who experienced a therapeutic response to sacral nerve stimulation, a decrease in activation in ROIs associated with urgency was seen. Specifically, brain activity decreased in the cingulate, insular, and frontal cortices and no new areas of increased activation were identified. Of great interest, this study also found an fMRI phenotype that was more likely to respond to targeted therapy ^26^. Women who responded to sacral nerve stimulation had greater brain activity noted prior to intervention in the cingulate, insular, and frontal cortices as well as the supplementary motor area and sensorimotor cortex compared with non-responders to therapy. We hypothesized that women who have increased brain activity in regions involved in bladder function may have more treatment targets available, thus explaining the improved treatment response in this subset of women.

Gill *et al.*, using 3 Tesla fMRI, demonstrated that sacral nerve stimulation in women with OAB who were responsive to therapy altered brain activity variably depending on stimulus intensity ^28^. The authors kept the neurostimulator on during imaging and used change in stimulus sensation as the study task. At subsensory levels, deactivation of the pons and periaqueductal gray (PAG) region occurred. The PAG is involved in distributing afferent input from the bladder to cortical regions as well as receiving cortical information to ultimately postpone or trigger urination ^29^. The authors hypothesized that this was the mechanism by which sacral nerve stimulation suppressed urgency.

Griffiths *et al.* found that patterns of brain activity in women with OAB who responded to biofeedback-assisted pelvic floor muscle training differed from those of women who did not ^29^. The authors used bladder filling-induced urgency as the study task and 3 Tesla fMRI. Similar to responders in the authors’ sacral nerve stimulation study above, responders were found to have activation of the cingulate, insula, and supplementary motor area prior to treatment and decreased activation in the cingulate and supplementary motor area after therapy. In non-responders, the medial prefrontal cortex was deactivated and changed little after pelvic floor muscle training. The authors suggest that prefrontal deactivation may be the mechanism by which pelvic floor muscle training is effective and that, owing to their starting point and a ceiling effect, non-responders in this study were unable to attain this ^29^. In a secondary analysis of these data, women with UUI who responded to biofeedback-assisted pelvic floor muscle training were found to have white matter damage in areas critical to bladder control ^30^. In women without damage in these specific areas, training was less effective, presumably because the cause of the UUI was not addressed. Together, these studies suggest at least two phenotypes of UUI with differential response to brain-targeted therapy such as biofeedback-assisted pelvic floor muscle training. Additional analysis of these data looking at functional connectivity of responders versus non-responders to biofeedback-assisted pelvic floor muscle training further supports the theory of different UUI phenotypes, one with a more brain-centric etiology ^31^.

Khavari *et al.* used 3 Tesla fMRI to look at brain activity in women with multiple sclerosis treated with intradetrusor botulinum toxin A for OAB symptoms ^32^. Mechanistically, botulinum toxin A inhibits neurotransmitter release from parasympathetic nerve terminals on the detrusor muscle and inhibits afferent signaling via purinergic receptors ^33, 34^. The authors used bladder filling-induced urgency and attempt to void as study tasks. They too found distinct effects on the activation pattern in brain regions known to be involved in bladder function. Interestingly, however, they found increased BOLD signal activity in the cingulate and prefrontal cortices, insula, pons, and thalamus after treatment. The authors accounted for this difference from the decreased BOLD activity seen in prior studies on antimuscarinics, neuromodulation, and pelvic floor muscle training by the fact that this population of neurogenic women has known central nervous system lesions (white matter plaque), which would be expected to alter functional connectivity, and was refractory to antimuscarinics and pelvic floor muscle training. In addition, after botulinum toxin A treatment, participants in this study required much larger volumes to register a full urge, which is the point when data analysis was performed. It is known that, in general, increased BOLD signals are seen at higher volumes and this study may have captured that effect. This study also demonstrated decreased BOLD signal activation in the amygdala and parahippocampal (fear and anxiety) regions following successful treatment with botulinum toxin A.

## Future directions

Emerging literature suggests that central pathophysiologic mechanisms may explain OAB and UUI. This is further supported by the age-related association of white matter disease severity and the extent of OAB and UUI ^35^. Effective treatment strategies for this condition, including antimuscarinic medications, sacral nerve stimulation, pelvic floor muscle training, and botulinum toxin, have demonstrated alterations in predesignated ROIs on fMRI, suggesting brain neuroplasticity. Altering abnormal brain region activity responsible for this condition may be effective targeted therapy. Phenotyping men and women with this condition to best allocate patients to successful treatment options would greatly advance the care of those with OAB and UUI. Further studies are needed to better understand the spectrum of phenotypic differences in OAB and UUI, differential responses to therapeutic interventions, and the predictive value of fMRI in individual classification versus group analysis. Investigations of alternative therapeutic mechanisms that alter brain activations in the identified ROIs and further elucidate the brain–bladder connection may offer novel targeted treatment approaches.
